# Surgery

**Published:** 1860-11

**Authors:** S. W. Gross

**Affiliations:** Lecturer on Surgical Anatomy and Operative Surgery, Philadelphia


### Bi-Monthly Abstracts;

OR, BI-MONTHLY NOVELTIES IN MEDICAL, PATHOLOGICAL, SURGICAL, OBSTETRICAL, PHYSIOLOGICAL, AND CHEMICAL SCIENCE.

### SURGERY.

BY S. W. GROSS, M.D.,

LECTURER ON SURGICAL ANATOMY AND OPERATIVE SURGERY, PHILADELPHIA.

I.—*On Iridectomy in Glaucoma.* By J. W. HULKE, F.R.C.S., (Medical Times and Gazette, July 21 and September 1, 1860,) and by W. BOWMAN, F.R.S., (Medical Times and Gazette, August 25, 1860.)

THE operation of iridectomy, consisting in the removal of a portion of the iris, was devised by Dr. A. von Graefe, of Berlin, to relieve permanently the intra-ocular pressure which always exists in the different stages of glaucomatous disease. This pressure, leading to great tension of the globe of the eye, gives rise to intense pain and a dilated and fixed condition of the pupils, which present a greenish hue, and is followed, sooner or later, by paralysis and congestion of the retina, excavation of the optic nerve, and inflammation, acute or chronic, of the component tissues of the eye, leading to their degeneration, and finally to complete loss of sight. The indications, therefore, are to restore the natural equilibrium of the circulation, and to prevent the destruction and compression of the retina, by removing the intra-ocular pressure. For this purpose, iridectomy, so warmly advocated by Graefe, Donders, Arlt, Bader, Hulke, Bowman, Critchett, and other experienced ophthalmic surgeons, is, in their opinion, the only resource, and they maintain that, until this process was adopted, all other forms of treatment proved useless in this disease. On the other hand, Hancock, Middlemore, Desmarres, and others, to accomplish the same object, drain off the excess of the fluid by means of puncturing the sclerotica; and between the surgeons of the two different schools a warm discussion has been, and is still being carried on, in relation to the merits of the two methods.

The operation of removing a portion of the iris is usually very simple. An incision is made at the junction of the sclerotica with the cornea, close in front of the iris, and a portion of this membrane is excised from its pupillary margin to its insertion, to the extent of a fifth or sixth of its circuit. The small quantity of blood which escapes into the anterior chamber must be removed either by gentle pressure or by means of a curette, and a wet cloth should be applied to the eye. Iridectomy may be performed either above or below, but the former is preferred on account of there being less disfigurement from the resulting coloboma of the iris.

[We have no experience with this operation, but we entirely agree with its opponents that it does not possess any of the advantages claimed for it by its inventor and his followers. How the removal of a portion of the iris acts otherwise than the more simple operation of paracentesis of the sclerotica, in relieving the intra-ocular pressure, we must confess we are unable to comprehend. To use the language of the caustic reviewer of the procedure, in an article entitled "Medical Epidemics—Glaucoma and Iridectomy," in the *Dublin Quarterly Journal of Medical Science* for August, 1860, and in whose views we, in the main, agree: "But then, we are told, it is not the mere letting out of the vitreous or aqueous fluid, but the cutting out of a portion of the iris, that relieves the pressure and effects the good. We are stupid enough not to see this in the same light as our neighbors. No doubt a wound made for the removal of a portion of the iris, even if none of that membrane remains in it, will not close so accurately nor heal so quickly as a puncture made by a broad needle; but if the pupil is free, the iris cannot by its presence or its bulk exercise any pressure on the optic nerve." In the second edition of his treatise on the diseases of the eye, Mr. Dixon, whose opinion is entitled to the greatest respect, refers to the operation in the following terms: "For myself, I may state that, although I could not recognize as sound the theory upon which the operation was brought forward as a cure for glaucoma, I tried it in a series of carefully-selected and well-marked cases of the following forms of disease: 'amaurosis with excavated optic nerve,' as Graefe has termed a peculiar morbid condition; chronic glaucoma, where the lens had not yet lost its transparency; and in cases of acute glaucoma, characterized by sudden impairment of sight, rapidly followed by inflammation of the eyeball, dilated and fixed pupil, severe neuralgia, and total loss of vision. In neither of the two first classes did I find—nor had I expected to find—any improvement to result. Nor in the third class was sight restored; but the inflammation seemed to be arrested, and the neuralgia was either very much lessened or it wholly ceased. I cannot, however, attribute this result to the removal of a portion of the iris, but merely to the evacuation of the aqueous humor through the large corneal wound."]

II.—1. *Inguinal Aneurism Cured by Digital Compression.* By W. C. NICHOLS, M.D. (New Orleans Medical and Surgical Journal, Sept., 1860.)

2. *Popliteal Aneurism Cured by Digital Compression.* By M. LAUGIER. (Gazette des Hôpitaux, and Lancet, June 9th, 1860.)

1. The first case occurred in an Irishman, twenty-eight years of age, who was admitted into the surgical ward of the Charity Hospital of New Orleans, under the charge of Dr. Stone, on account of a large aneurism situated in the right inguinal region, above Poupart's ligament. The tumor was the seat of great pain, and by thrusting the finger deeply into the iliac region, over the course of the artery, its pulsation could be arrested.

The compression was conducted by Dr. Nichols, assisted by a number of physicians and students, who began the treatment by pressing the thumb against the neck of the sac, on the seventeenth of February, at five o'clock P.M. Some inconvenience was experienced in effecting the purpose, on account of the tenderness of the parts, but, the patient being calmed by opium, the compression was continued; and in thirty hours the pulsation in the tumor had ceased. As a matter of precaution, however, the pressure was kept up ten hours longer, when it was discontinued, a cure having been effected. Three months subsequently the patient presented himself at the hospital, when the tumor was found to have contracted to the size of a walnut.

[This is the fourth case in which digital compression on the external iliac for inguinal aneurism has been tried, and is the only one that has resulted successfully. The other three cases failed, on account either of the great fatigue experienced by the assistants, or from the pressure being unbearable. Dr. Nichols has, therefore, occasion to congratulate himself on the success of his treatment.]

2. M. Laugier instituted digital compression on the femoral artery for a popliteal aneurism, the size of a turkey's egg, in a man forty-eight years of age. In twelve hours concentric indurations, from clots, had formed; the tumor was very hard in forty-eight hours, and for seventy-two hours the compression was continuous. After this time it was carried on intermittently every fifteen minutes. After eighty hours the tumor was solid and very hard, and only a slight pulsation, without any expansion, could be felt at the upper part of the sac.

III.—*Dislocation of the Scapular End of the Clavicle Treated by Wiring.* By J. T. HODGEN, M.D., Professor of Anatomy in the Missouri Medical College. (Kansas City Medical and Surgical Review, Sept., 1860.)

James O'Reardon, on the 20th of May, 1860, fell from a height of fourteen feet upon his left shoulder, severely contusing the soft parts. The scapular extremity of the clavicle was completely displaced from the acromion, forming a marked prominence, and occupying a position two inches upward and backward from the acromial facet. The scapula and arm hung closely along the ribs. To maintain the parts in apposition, the ordinary apparatus for fractured clavicle was applied, and direct pressure, by means of a firm compress, was made upon the acromio-clavicular junction. This, with a variety of other bandages, was employed without success for two weeks, when, the swelling and inflammation having disappeared, wiring the bone was determined upon. The dislocation being reduced by an assistant, an incision was made over the bones and they were accurately brought together by means of twelve strands of fine iron wire, twisted into a cord as large as a crow-quill. No untoward symptom followed the operation, and on several occasions, the wire becoming loose, it was tightened. On the twenty-fifth day the wire was removed, and on the twelfth of August the external wound had healed, but the clavicle was not accurately in place, being about half an inch too high and too far back.

[That this method is the best adapted for maintaining the dislocated bones in apposition, no one can doubt; and Dr. Hodgen remarks: "So far as my knowledge goes, this operation has never been performed." He will, however, find in relation to the treatment by the wire, that he has been anticipated by Professor Gross, who has for years inculcated it in his lectures. In his *System of Surgery*, under the head of dislocations of the clavicle, he refers to it in the following paragraph: "Seeing how difficult it is to keep these various dislocations of the clavicle reduced, I should not hesitate, if an opportunity arose, to fasten the ends of the bones with a silver wire, inserted subcutaneously, and retained until reunion occurred. The operation could be easily executed, and would not be likely to cause any bad effects."]

IV.—*On Excision of the Knee-Joint.* By E. KRACKOWIZER, M.D. (American Medical Times, Sept. 15th, 1860.)

In this paper, read before the New York Academy of Medicine, the author discusses more particularly the mortality attending excision of the knee-joint, and the objections which have been urged against the operation. He has collected two hundred and thirty-three cases, which give a result of sixty-three deaths and twenty subsequent amputations, the rates of mortality being twenty-seven in one hundred; or, if we count the subsequent amputations, the ratio of failures will be thirty-six in one hundred. Of these cases thirteen occurred in the practice of American surgeons, four resulting in death, and two being under treatment, at the time of the report, with a prospect of recovering a good limb.

V.—*Fracture of both Femurs by Muscular Spasm.* By FREDERIC D. LENTE, M.D. (American Medical Times, No. 3, vol. i.)

Dr. Lente reports a case of this unusual accident, occurring in a lad twelve years of age, who had, from the age of fifteen months, been the subject of epilepsy. For several years past he had suffered from partial hemiplegia of the right side, and from convulsions of a very severe nature and of a tonic variety. The case, the history of which we append below, is rendered the more interesting from the fact that both bones were fractured during a tonic spasm, not by a sudden jerk, a point which renders it perhaps unique. There was no evidence of fragility of the bones, although they were smaller than is usual in children of the age of the patient:—

"On the 10th of April, 1859, his spasms had been recurring every few minutes with great violence; during one of them, while he was held in bed by one of the family by the arms and shoulders—a number of others being in the room—a loud snap was heard by all present. It was thought that the hip had slipped out of place, and, upon examination by the parents, what they supposed to be an extensive swelling was perceived at the upper part of the left thigh. Upon being called in soon after, I recognized it at once as a fracture of the femur; its seat was the junction of the upper with the middle third of the bone. It is stated by the friends that, at the instant of its occurrence, the thigh was flexed with great force by the intensity of the spasm on the pelvis, and the fracture was evidently effected by the powerful action of the flexor muscles of the thigh. Assisted by Dr. Richardson, I administered ether, extended the limb, and applied a thick pasteboard splint to the thigh, carrying the bandage around the pelvis, the only idea being to secure union with the least possible inconvenience to the patient, with little regard to shortening.

"June 1st.—Union of the fracture is firm, with considerable bowing and shortening, as was anticipated. A pasteboard splint encircling the thigh, and coated with shellac to prevent injury from urine and other fluids, is still kept on to prevent a repetition of the fracture, as he still has the convulsions.

"Dec. 13th.—The right femur was fractured to-day in precisely the same manner as was the left, and it was put up in the same manner.

"Jan. 29th.—The patient's health has been gradually failing since the occurrence of the last fracture, and to-day he died from gradual exhaustion. No attempt at union of the fracture has taken place. No autopsy could be procured."

VI.—*Excision of the Elbow-Joint and of the Entire Ulna.* By GEORGE WILLIAMSON, M.D. (Notes on the Wounded from the Mutiny in India.)

H. W., twenty-six years of age, was admitted into the Hong-Kong general hospital, in August, 1857, on account of acute pleurisy and hepatitis. In a few weeks a large abscess near the left elbow-joint was evacuated, and from this time the ulna became necrosed, permanently enlarged, and a few spiculæ of bone came away. A year subsequently he was received into the Fort Pitt Hospital, with numerous sinuses along the inner side of the ulna, and a probe could be introduced into the elbow-joint. His general health was good.

On the 30th of August, 1858, the whole of the ulna, an inch and a half of the humerus, and the head and neck of the radius, close to the tubercle, were removed by an incision along the inner and posterior side of the forearm. No vessels required ligation, and the arm was supported in a semi-flexed position on a gutta-percha splint. On the sixth day the greater part of the wound had united by the first intention, and by the twelfth day the union was complete. At the time of the report, four months after the operation, the man "could bend his forearm, raise his hand behind his head, and lift a twenty-eight pound weight from the ground. He could also pronate and supinate the hand; there was no anchylosis of the wrist-joint, and he could use his fingers well."

VII.—*Traumatic Inflammation of the Sinuses of the Dura Mater.* By PROFESSOR PITHA. (Oesterreich Zeitschrift für praktische Heilkunde, and Prager Vierteljahrschrift, I., 1860.)

A hussar, twenty-six years of age, received several sabre wounds on the head, one being situated on the forehead, another on the vertex, a third on the occipital protuberance, and a fourth behind the ear; the three former implicating only the skin. He labored under all the symptoms of concussion of the brain for nearly four weeks, and on the twenty-eighth day he was attacked with febrile disturbance and extreme restlessness, and the discharge from the wound behind the ear increased in quantity. On the thirty-second day chills, vomiting, and bronchial respiration supervened, and he was much prostrated. Twelve days subsequently he lost his sight, the globe of the right eye being very prominent and tense, and the pupil fixed and slightly dilated, and the conjunctiva œdematous. On the following morning death took place, the left eye having assumed the same appearances as the right.

On a post-mortem examination the wounds of the scalp were found to be completely cicatrized. The mastoid process of the temporal bone had sustained a loss of substance about the size of a bean, and the bottom of the wound was covered with grayish pus and fine granular exudation. For about half an inch in circumference around the erosion the bone was rough, the roughness extending to the mastoid vein communicating with the lateral sinus, the orifice of which was three-fourths of a line wide, and covered with a layer of lymph. The substance and membranes of the brain were normal, with the exception of that portion of the dura mater lining the middle fossa of the skull, which was covered with a thin layer of lymph. The left lateral, superior petrosal, circular, and cavernous sinuses, up to the ophthalmic vein, were filled with a thick, yellowish-white pus. The corresponding sinuses on the right side, and the two ophthalmic veins were occupied with coagula, and contained here and there some thick purulent matter. Both lungs were filled with circumscribed abscesses.

VIII.—*Dislocation of the Crystalline Lens into the Anterior Chamber of the Eye during a Fit of Sneezing.* By JABEZ HOGG, Esq. (London Lancet, No. 24, vol. i., 1860.)

Instances of dislocation of the crystalline lens are so rare that they deserve to be placed on record, and this case is the more interesting from the fact of the accident being produced during a fit of sneezing. The causes of this displacement are usually injury to the globe of the eye, falls upon the feet or on the head, or blows inflicted on any portion of the head; or it may take place without any assignable cause. This is the first instance, however, which has been produced in so singular a manner.

The patient was a German, thirty-six years of age, who had always been myopic, and whose sight had been weak for several years. Two months previous to the occurrence, his vision became very acute, and, on the eighteenth of April, after a fit of sneezing, he suddenly became blinded, and found that he was very near-sighted. On examination, the lens was seen in the anterior chamber, producing considerable concavity of the iris. The pupil was kept dilated by atropine, and on the eighth day inflammation with photophobia set in, and during the night he suffered intense agony, which could not be relieved by leeching, morphia, or chloroform. The following day Mr. Hogg extracted the lens, and the patient was immediately freed from pain. On the thirteenth of May he was discharged, and has now quite recovered, the sight remaining good.

IX.—*Report of Cases of Vesical Calculus Operated on during the Years* 1858 *and* 1859, *in the Norfolk and Norwich Hospital.* By MR. CHARLES WILLIAMS. (London Lancet, August 18, and September 1, 1860.)

Twenty-two cases of stone in the bladder have been operated upon in this hospital, during the past two years, by Drs. Crosse, Firth, Nichols, and Cadge, and, with one exception, all were attended with success. The fatal case occurred in a child, eighteen months old, upon whom the median operation had been performed, and who died a few hours afterwards. The ordinary lateral operation was performed in thirteen, the median in eight, and in the case of a female, the stone was extracted by dilatation. The ages of the patients varied from eighteen months to seventy-four years, fourteen being fifty and upwards, and eight below that period of life. In fifteen the calculi were composed of lithic acid, in four of oxalate of lime, and in three of phosphates. In the case of a pensioner, seventy years old, there was a remarkable tendency to the formation of calculi, at one time composed of uric acid, at others of phosphates. He had been cut in the hospital on two occasions, and so often previously that he was unable to reckon the number.

It is interesting to note that the Norfolk and Norwich Hospital contains the largest collection of human urinary calculi in the world, exclusively the produce of a single establishment. The number that have been removed amounts to nine hundred and eighty-two, and the museum also contains five hundred and forty-two other specimens, presented by different surgeons. Since the establishment of this charity, in July, 1772, to the end of 1859, eight hundred and sixty-three patients have been operated upon by the following methods:—

| | |
|---|---|
| Lateral operation | 803 |
| Dilatation in female | 41 |
| Lithotrity | 11 |
| Median operation | 8 |
| | 863 |

Of this whole number, the recoveries were seven hundred and fifty-five; the deaths, one hundred and eight; the proportion being 1 in 7·99. Of the eight hundred and three cases in which the lateral operation was performed, the proportion of deaths was 1 in 7·649. Of the forty-one females, the proportion of deaths was 1 in 20·5; two cases having resulted fatally.

X.—*On Iritis as it Occurs in Syphilitic Infants.* By JONATHAN HUTCHINSON, Esq. (Med. Times and Gazette, vol. ii., 1860, No. 524.)

This infrequent but destructive and insidious disease has either been greatly overlooked, or its study has been neglected by the profession at large, and it is with a view to add to our correct knowledge upon the subject that Mr. Hutchinson has collected into a tabular statement all the examples of hereditary syphilitic iritis which he has been able to find recorded. After a careful search through the journals and elsewhere, he has discovered but twenty-one cases, and from the series he deduces the following conclusions:—

1. That the subjects of infantile iritis are much more frequently of the female than of the male sex, as shown by the fact of fourteen of the infants being girls and five boys. In two cases no information is given of the sex.

2. That syphilitic infants are most liable to suffer from iritis at about the age of five months. The youngest patient was seven weeks of age; the oldest, sixteen months.

3. That syphilitic iritis in infants is often symmetrical, but quite as frequently not so. In nine cases of the series both eyes were affected, and in eleven the inflammation was limited to one; but in the latter class it is quite probable that the other eye suffered subsequently.

4. That iritis, as it occurs in infants, is seldom complicated, and is attended by but few of the more severe symptoms which characterize the disease in the adult. Sclerotic congestion and pain were absent in more than one-half the cases, and in but very few was there any marked photophobia, or haziness of the cornea.

5. Notwithstanding the ill-characterized phenomena of acute inflammation, the effusion of lymph is usually very free, and the danger of occlusion of the pupil great.

6. Mercurial treatment is most signally efficacious in curing the disease, and, if recent, in procuring the complete absorption of the effused lymph. In all cases where this remedy had a fair chance, the disease promptly yielded to it.

7. Mercurial treatment previously adopted does not prevent the occurrence of this form of iritis.

8. The subjects of infantile iritis, though often puny and cachectic, are also often apparently in good health; showing that the more ill-nourished are not the most prone to be attacked.

9. That infants suffering from iritis almost always show one or other of the well-recognized symptoms of hereditary taint. In two instances only were there no other specific symptoms of constitutional taint; but in the remaining cases, the following symptoms were present at the outbreak of iritis: Psoriasis of the general surface in nine cases; a papular rash in two; psoriasis palmaris in one; erythema marginatum in two; peeling of the skin in one; falling of the eyelashes and tinea tarsi in two; snuffles in ten; sore mouth and aphthæ in four; and condylomata of the anus in five.

10. Most of those who suffer from syphilitic iritis are infants born within a short period of the date of the primary disease in their parents. "In one instance the mother had contracted primary syphilis from her husband only three months before the birth of the infant. In another, the interval was four, and in a third, six months. In five, about a year had probably elapsed; and in another five, at least two years. In two, judging by the fact that the mother had previously borne a number of children, some of which had died with suspicious symptoms, the date of the original disease in the father could not be placed nearer than six or seven years. This calculation quite accords with what is observed in the iritis of adults, which, in a great majority of instances, is a secondary—and not a tertiary—symptom."

